# Aluminium in the Human Brain: Routes of Penetration, Toxicity, and Resulting Complications

**DOI:** 10.3390/ijms24087228

**Published:** 2023-04-13

**Authors:** Łukasz Bryliński, Katarzyna Kostelecka, Filip Woliński, Piotr Duda, Joanna Góra, Michał Granat, Jolanta Flieger, Grzegorz Teresiński, Grzegorz Buszewicz, Ryszard Sitarz, Jacek Baj

**Affiliations:** 1 Student Scientific Group, Department of Forensic Medicine, Medical University of Lublin, ul. Jaczewskiego 8b, 20-090 Lublin, Poland; 2 Student Scientific Group, Department of Anatomy, Medical University of Lublin, ul. Jaczewskiego 4, 20-090 Lublin, Poland; 3 Department of Analytical Chemistry, Medical University of Lublin, Chodźki 4A, 20-093 Lublin, Poland; 4 Department of Forensic Medicine, Medical University of Lublin, Jaczewskiego 8b, 20-090, Lublin, Poland; 5 I Department of Psychiatry, Psychotherapy and Early Intervention, Medical University of Lublin, 20-059 Lublin, Poland; 6 Department of Anatomy, Medical University of Lublin, ul. Jaczewskiego 4, 20-090 Lublin, Poland

**Keywords:** aluminium, human brain, Alzheimer’s disease, autism spectrum disorder, alcohol use disorder, multiple sclerosis, Parkinson’s disease, dialysis encephalopathy

## Abstract

Aluminium (Al) is the most ubiquitous metal in the Earth’s crust. Even though its toxicity is well-documented, the role of Al in the pathogenesis of several neurological diseases remains debatable. To establish the basic framework for future studies, we review literature reports on Al toxicokinetics and its role in Alzheimer’s disease (AD), autism spectrum disorder (ASD), alcohol use disorder (AUD), multiple sclerosis (MS), Parkinson’s disease (PD), and dialysis encephalopathy (DE) from 1976 to 2022. Despite poor absorption via mucosa, the biggest amount of Al comes with food, drinking water, and inhalation. Vaccines introduce negligible amounts of Al, while the data on skin absorption (which might be linked with carcinogenesis) is limited and requires further investigation. In the above-mentioned diseases, the literature shows excessive Al accumulation in the central nervous system (AD, AUD, MS, PD, DE) and epidemiological links between greater Al exposition and their increased prevalence (AD, PD, DE). Moreover, the literature suggests that Al has the potential as a marker of disease (AD, PD) and beneficial results of Al chelator use (such as cognitive improvement in AD, AUD, MS, and DE cases).

## 1. Introduction

Aluminium is the third most common element and the most ubiquitous metal of the Earth’s crust, constituting over 8% of its mass [1]. However, it is not essential for human metabolism [2,3], and adversely it can be toxic for the human organism, including the brain [4,5]. This fact is worrying, considering that we live in the ‘Aluminium age’, where exposure to this extensively used metal is inevitable and burgeoning [6]. Absorbed via various routes, Al can display toxic properties, some of which can be associated with the pathogenesis of Parkinson’s disease (PD), Alzheimer’s disease (AD), autism spectrum disorder (ASD), alcohol use disorder (AUD), multiple sclerosis (MS), and dialysis encephalopathy (DE). Therefore, according to the literature sources, Al concentration could be used as a marker of certain diseases (AD, PD), and possible benefits from the use of Al chelators (AD, AUD, MS, DE) are possible.

In this review, we aimed to collect data regarding the sources of exposure to Al, routes of its absorption into the body, and the molecular mechanism of its toxic effects in the pathogenesis of several neurological diseases. We performed the literature review using PubMed and UpToDate based on international papers in the English language. Articles published between 1976 and 2022 were considered. We used the following phrases: Aluminium, Aluminium exposure, Aluminium environment, Aluminium in brain, Aluminium gastrointestinal absorption, Aluminium lung absorption, Aluminium intake, Aluminium skin absorption, Aluminium antacids, Aluminium vaccines, Aluminium drugs, Aluminium excretion, Aluminium drinking water, Occupational exposure to Al, Aluminium toxicity, Parkinson’s disease, Alzheimer’s disease, autism spectrum disorder, alcohol use disorder, multiple sclerosis, and dialysis encephalopathy. The main focus was on the articles from international scientific journals available through Pubmed, UpToDate, and Google Scholar. Guidelines from the World Health Organization (WHO), Agency for Toxic Substances and Disease Registry (ATSDR), EFSA, and SCCS were used. Finally, the results of 125 articles, which described Al sources and ways of elimination, toxic mechanisms, and the role of Al in the pathogenesis of several diseases: AD, ASD, AUD, MS, PD, and DE, were collected. Thus, the current knowledge related to clinical trials, systematic reviews, meta-analyses, and case reports was taken into account.

### 1.1. Al Sources and Elimination

The primary natural sources of Al are rocks (such as bauxite, silicates, and cryolite) and, to a lesser extent, surface, and subsurface waters and soils, to which Al migrates as a consequence of natural weathering processes and volcanic activity [7,8,9]. These phenomena belonging to the ‘geochemical cycle’ were never a source of a biologically reactive Al ‘throughout biochemical evolution’, which justifies its non-essentiality for living organisms [6]. The lithosphere-to-biosphere transfer, which depends on the incorporation of Al into the ‘biogeochemical cycle’, is predominantly the effect of either indirect or direct human activity [6,10]. The first involves the influence of acid rains releasing Al ions into the environment, and the other consists of Al extraction from biologically inert ores for industrial purposes [10]. Among the latter, we might distinguish materials used for vehicle and airplane production, construction and building materials, packaging, electrical devices, foods, drinking water, cosmetics, personal care products, medicaments, and a variety of utensils [7].

**Figure 1:**
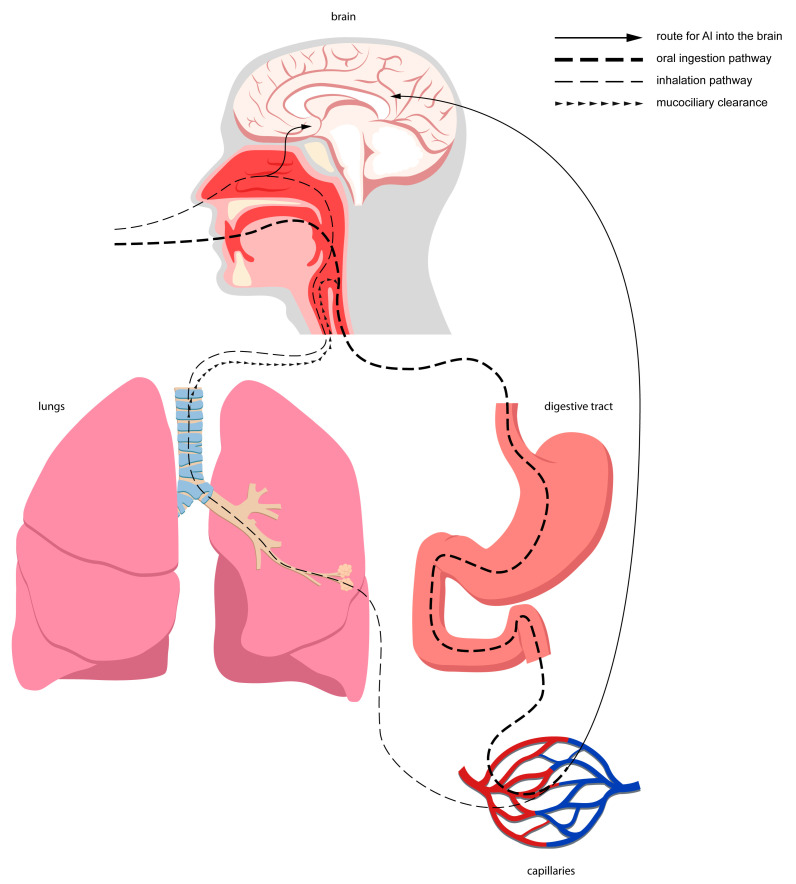
Main routes for Al into the brain. Red capillaries symbolize arterioles, while blue capilarries symbolize small veins.

Nevertheless, the above-mentioned sources vary significantly in terms of Al content and route and degree of absorption of this element. Moreover, each individual is differently exposed to these sources, which translates into the distinct burden of biologically reactive Al that could impact human health. The main penetration routes for Al into the human organism are oral intake (either from foods or beverages) and inhalation. Furthermore, the digestive tract is the leading Al absorption site for the general population [4,11]. It is worth mentioning that aspired Al, which is available mostly for occupationally exposed populations, for instance, during mining and processing of Al ores, welding, cutting, etc. [4], could be absorbed into the bloodstream either directly through the lung tissue [11] and respiratory epithelium of the nasal cavity or enter the gastrointestinal tract via mucociliary clearance and swallowing. The nasal cavity also contains the olfactory epithelium, which makes up the direct pathway for Al into the brain [6]. Figure 1 shows the main routes for Al into the brain.

Considering food ingestion, it constitutes a source of about 50% of Al’s Tolerable Weekly Intake (TWI) settled by the European Food Safety Authority (EFSA) [12]. Among food sources, vegetables contribute to the most Al exposure [13] (see Table 1 and Table 2).

It should be kept in mind that the presence of Al in foods is a result of both original content (from environmental sources and food additives) and the following interaction with Al-containing materials used for food packaging and cooking [4,10]. Drinking water contributes to the total oral exposure to Al, usually less than 5% [25]. Al content in drinking water is a sum of Al present in all natural waters and the one coming from Al salts used for water treatment processes [9]. The usually achievable Al concentrations in drinking water (0.1–0.2 mgAl/L) are close to the acceptable levels (0.05–0.2 mgAl/L) settled independently by many countries [9,25]. It is worth noting that Al is absorbed in merely about 0.1% and 0.3% of food and drinking water, respectively [4]. The Al uptake itself depends on several factors listed in Table 3.

As a consequence of using widespread over-the-counter antacids, Al ingestion might exceed that in food and beverages by over 100-fold [26], although the absorption is usually in the range of 0.01–1%. It was estimated that orange juice could increase Al absorption from antacid drugs by 8-fold and that citric acid increases the intake by up to 50-fold [27].

To a lesser degree, Al might enter the system through other routes. Al exists in thousands of formulations of cosmetics and personal care products, such as antiperspirants, lipsticks, liquid makeup foundations, toothpaste, etc. There are very few studies concerning Al absorption through the skin [28]; however, the Scientific Committee on Consumer Safety (SCCS) recommended safe limits for sprayable and non-sprayable Al-containing cosmetic products [29]. Among pharmaceuticals, apart from previously mentioned antacids, Al is also functioning as a vaccine adjuvant. However, it should be taken into account that the vaccination itself is a rather sporadic event and that the Al content in a single vaccine dose is limited to 1.25 mg. It was stated that the risk of Al toxicity for the most vulnerable group, which is infants, and therefore for the general population, is minimal compared to the benefits related to the vaccination itself [30] and that there are no indications related to neurotoxicity for the elimination of Al from the vaccines [31]. In addition, formerly, patients with chronic kidney disease formed a substantial group exposed to Al due to contamination of dialysis water with Al compounds and ingestion of Al-containing phosphate binders. Currently, it is no more a common issue in many countries due to the removal of Al from the water used for dialysis and new phosphate binders free from Al [32].

Al is excreted from the body through numerous routes, depending on whether it has been absorbed into the bloodstream or where it had been deposited in the organism. The absorbed fraction is eliminated (as the Al ion) in 95% with urine. Unabsorbed Al located in the gastrointestinal tract, either ingested or coming from the aforesaid mucociliary clearance, is excreted via the feces [6,12]. Other possible routes of Al elimination comprise the skin, hair, sebum, nails, sweat, semen, milk, and bile [4,6].

### 1.2. Mechanisms of Aluminium Toxic Effects

Although we know for sure that Al accumulates in the brain [33,34], it is not fully understood how it reaches it. Possibly, similar to other nonessential metals, it hijacks physiological transportation and absorption mechanisms [35]. The major fraction of Al (about 90%) after absorption is bounded by serum transferrin (Tf), which is also responsible for the transportation of iron (Fe) cations. This protein can intercede in the transportation of Al through the blood–brain barrier (BBB) by means of transferrin receptor (TfR)-mediated endocytosis. Most of the remaining 10% circulates as Al citrate, which is much more prominent in cerebrospinal fluid (CSF). This suggests the existence of yet another transportation mechanism independent of Tf [36]. In addition, Al is capable of selectively increasing the rate of diffusion across BBB [37]. It was demonstrated that some blood vessels display a greater affinity for Al accumulation than others. Those include brain arteries lined with human brain microvessel endothelial cells, especially the posterior cerebral artery that supplies the hippocampus. Besides the hippocampal area, Al is mostly deposited in the cerebellum and cortex [36,38]. Additionally, Al can probably reach the brain directly through the continuity of the olfactory epithelium, the olfactory nerve, and the olfactory bulb [6].

Considering the aforementioned toxicity, Al cations and their compounds can disrupt crucial cell functions and processes. Thus, the effects of exposure to Al are visible on a molecular and systemic level. The neurotoxic features of chronic Al toxicity are well-documented [39]. In the mammalian brain, intracisternal [40] and oral [41] Al supply results in a neurofibrillary degeneration pattern that could resemble the neurofibrillary tangles (NFTs) present in Alzheimer’s disease (AD) patients [42]. However, Oshima et al. [43] proved that after chronic oral Al ingestion, promoted tau aggregation, apoptosis, and neurological dysfunctions were only observed in transgenic mice already having tau aggregation, contrary to wild-type mice.

When confronted with Al cations, protein polypeptides can either denature or undergo conformational or structural alternation, as in β-amyloid plaques. Moreover, Al blocks the proteolytic degradation of amyloid, enhancing its deposition and aggregation [4,28,44,45,46] and increasing its permeability in the striatum and thalamus [47]. Furthermore, it was demonstrated that Al promotes phosphorylation and aggregation of phosphorylated proteins such as Tau protein. Additionally, according to some studies, it increases the expression of the precursor amyloid protein (APP), β-40, and β-42 fragments and prolongs Aβ-42 half-life in blood [47,48,49], though results have not always been consistent [28,36]. Al can influence the activity of important neuronal enzymes such as Alkaline Phosphatase and Acetylocholinesterase as well as decrease neurotransmission. Moreover, it increases the expression of Cyclin D and Cathepsin D, which are essential cell cycle proteins [34,36]. Several Al compounds exerted neuronal and glial apoptosis in hippocampal cell cultures [50]. On the contrary, in other studies involving mice, excessive oral Al supply did not increase either Aβ or tau protein accumulation [51] or altered spatial learning and memory with no effect on neurogenesis [52]. Noticeably, Al-maltolate-treated aged rabbits are suggested as the best animal models for Al-induced AD [53]. 

Al interferes with the energy metabolism of hepatocytes by impeding ATP production, inhibiting glycolysis and the Krebs cycle, and promoting protein and lipid oxidation. Additionally, it damages metal processing causing Fe overload, which boosts oxidative stress and, as a result, causes DNA damage and cell death [4,36]. Moreover, Al causes apoptosis of lymphocytes (immunosuppression) and erythrocytes. It can affect bone mineralization and formation by increasing osteoclast activity, decreasing osteoblast function (via interacting with the Wnt/β-catenin signaling pathway, bone morphogenic protein 2 (BMP-2) signaling pathway, and transforming growth factor-beta 1 (TGF-β1) expression), and inhibiting vitamin D biological properties (for example by blocking stimulation of synthesis of osteocalcin in osteoblasts). It is worth mentioning that the aforesaid BMP-2 and TGF-β1 pathways are essential for proper cartilage formation. Some studies linked exposure to Al with hypertension, ischemic strokes, and endocrine disruptions (Al affects the secretion of parathormone, testosterone, luteinizing hormone, follicle-stimulating hormone, estradiol, norepinephrine, cortisol, thyroid hormones, and insulin). Furthermore, Al concentration in the cell nucleus negatively impacts proliferation and differentiation, and thus it is considered genotoxic. This may be connected with the metastatic process of breast cancer (activating matrix metalloproteinase 9 (MMP9) and matrix metalloproteinase 14 (MMP14)). Lastly, Al is considered a proinflammatory and proapoptotic agent, up-regulating various cytokines such as Interleukin-1β and tumor necrosis factor α (TNFα) in numerous tissues [4,28,54,55,56,57]. 

Among the systemic effects of Al, one may mention the following:

- Pulmonary lesions—Al has been connected with disorders such as granulomatosis and fibrosis of the lungs, pneumonia, pulmonary edema, and pulmonary alveolar proteinosis. Possibly it is also connected with asthma;
- Cardiovascular effects—in the case of Al phosphide intoxication, myocarditis, hypokinesia, left ventricular thrombosis, and stroke were reported. Among pregnant women, greater Al hair concentration correlated with a higher incidence of congenital heart defects in their offspring;
- Hematologic effects—include depressed erythropoiesis and subsequent anemia;
- Musculoskeletal effects—exposure to Al can cause macrophagic myofasciitis associated with arthromyalgia and chronic fatigue syndrome. Osteoporosis, rickets, exostosis, osteodystrophy, and osteitis fibrosa are also triggered by this metal;
- Neurological effects—higher Al hair concentrations were connected with dialysis encephalopathy (DE), Parkinson’s disease (PD), amyotrophic lateral sclerosis (ALS), multiple sclerosis (MS), and autism spectrum disorder (ASD) [4,56,58,59].

## 2. Alzheimer’s Disease

AD is the most common cause of dementia, contributing to 60–70% of its cases [60]. Between 2011 and 2050, the number of AD patients is predicted to rise threefold, with an estimated over 100 million patients by 2050 [61]. Regarding anatomopathological analyses, Al was shown to appear in the core of senile plaques within the hippocampus and temporal lobes in AD patients [62]. Compared with non-demented patients, Al concentration in brain samples of AD sufferers was reported higher in the hippocampus [63] and temporal gyri [64], as compared with non-AD patients (Table 4). However, Akatsu et al. [65] found no statistically significant differences between AD patients and non-demented patients in the hippocampus and amygdala. Virk et al. [66], in a 2015 meta-analysis, compared the levels of Al in the brain, serum, and CSF of AD and non-AD individuals. AD patients had higher Al levels in all of the analyzed tissues, and those authors suggested that plasma Al levels could be an early marker of AD development [66]. 

However, despite many reports on AD-promoting action and being the most widely studied environmental agent in the pathogenesis of AD [66], the link between Al and AD remains a source of intense scientific debate [39,67,68]. The majority of epidemiological studies suggest a link between AD and chronic exposure to Al [39], specifying drinking water and occupational exposure to Al as the two most common sources of chronic exposure to Al [68]. 

In 2016, Wang et al. [68] published a meta-analysis including 10567 participants from eight epidemiological studies published up to June 2015. The chronic exposure to Al via drinking water or the subject’s occupation was associated with an increased risk of AD development, with OR of 1.95 (95% CI, 1.47–2.59) and 1.25 (95% CI, 0.80–1.94), respectively [68]. However, a 2015 meta-analysis found no link between AD and occupational exposure to Al among 1056 participants [69]. In two large prospective studies, Rodeau et al. [70,71] investigated the links between Al and silica exposure on the development of dementia and AD among 3777 subjects aged at least 65 years (PAQUID cohort). The second study comprised additional 400 subjects from the ALMA+ cohort, but no data regarding AD were provided in this group. In the first study, evaluation was made among 2698 patients after a mean follow-up of 5.9 years [70], while, in the second study, 1677 subjects were analyzed after a mean of 11.3 years [71]. In the first study, the authors found an epidemiological link between high water Al concentration (at least 0.1 mg/L) and higher AD prevalence with an RR of 2.20 (95% CI, 1.24–3.84) [70]. Similarly, in the second study, the authors found an epidemiological link between high daily water Al consumption (at least 0.1 mg/day) and higher AD prevalence (RR of 3.35 with 95%CI of 1.49–7.52) [71]. 

Al hypothesis in AD development is linked with the therapeutic use of Al chelators. The first widely used Al chelator was deferoxamine (DFO), and despite reported clinical usefulness in AD, its adverse effects and administration via long-lasting injection limited its usability [72]. However, the recent literature review by Agrawal et al. [73] on intranasal AD drugs pointed clearly to intranasal DFO as a potential candidate for AD treatment. Other metal chelators that were evaluated in AD treatment are silicon (Si) compounds, which are natural antagonists of Al [74]. The first study that showed reduced Al burden in AD after Si-rich mineral water drinking was published in 2006 by Exley et al. [75]. Among subsequent studies, Davenward et al. [74] tested the impact of 12-week Si-rich mineral water drinking treatment on Al-body-burden among 15 AD and 14 non-AD participants. Such therapy reduced the body Al burden in both groups and improved cognitive outcomes in three of the AD sufferers [74]. Results of two above-mentioned epidemiological studies by Rondeau et al. [70,71] showed that high water silica concentration (at least 11.25 mg/L) [70], or 10 mg/day increase in water silica [71] were associated with lower AD prevalence with RR of 0.69 (95%CI, 0.52–0.94) [70] and 0.88 (95% CI, 0.79–0.99) [71], respectively. AD was also suggested to be linked with Al-containing antacid drugs. However, in a meta-analysis comprising seven case-control and two cohort studies, regular Al-containing antacids use was not associated with AD [67].

**Table 4:** The concentration of aluminium in the tissues of patients with particular neurological diseases.

Disease	Tissue	Level/Concentration in Tissue of the Patients	Level/Concentration in Tissue of Control Group	Additional Information	Reference
AD	hippocampus	0.000357 mg/g	0.00009 mg/g	The differences in the concentration of Al between patients with AD and the control group were statistically significant.	[63]
AD	the temporal lobe of the brain	0.0019–0.0168 mg/g	0.00016–0.0018 mg/g	The differences in the concentration of Al between patients with AD and the control group were statistically significant.	[64]
ASD	occipital lobe; frontal lobe; temporal lobe; parietal lobe;	0.00382 mg/g; 0.00230 μg/g; 0.00279 mg/g; 0.00382 mg/g;	N/A	-	[76]
ASD	the temporal lobe of the brain	0.0009–0.0016 mg/g	0.00016–0.0018 mg/g	The authors found no association between ASD and Al concentration in temporal gyri.	[64]
AUD	total brain content; thalamus; inferior longitudinal fasciculus; insula; superior longitudinal fasciculus;	0.00159 mg/g; 0.00405 mg/g; 0.00348 mg/g; 0.00241 mg/g; 0.00108 mg/g;	All control samples displayed Al content below detection limits.	In this research, authors also showed that the Al levels in the liver displayed no significant difference between AUD and control subjects.	[77]
MS	brain	0.0012 mg/g	0.0006 mg/g	The differences in the concentration of Al between patients with MS and the control group were statistically significant.	[78]
MS	scalp hair samples	0.00376 mg/g	0.00449 mg/g	The differences in the concentration of Al between patients with MS and the control group were statistically significant.	[79]
MS	urine	7.51 μM	0.35 μM	The differences in the level of Al between patients with MS and the control group were statistically significant.	[80]
DE	brain	0.00159 mg/g	0.0044 mg/g; 0.0027 mg/g;	Mean brain concentrations of Al were 0,00159 mg/g, 0,0044 mg/g, and 0,0027 mg/g among patients dying from dialysis encephalopathy, among the dialyzed control group, and among uraemic patients who were not dialyzed, respectively. The differences in the concentration of Al between patients with DE and the control groups were statistically significant.	[81]
DE	muscle	14.8 ppm	1.2 ppm	The differences in the concentration of Al between patients with DE and the control group were statistically significant.	[82]
DE	trabecular-bone	98.5 ppm	2.4 ppm	The differences in the concentration of Al between patients with DE and the control group were statistically significant.	[82]
DE	brain grey-matter	25 ppm 6.5 ppm	2.2 ppm	Mean brain concentrations of Al were 25 ppm, 6.5 ppm, and 2.2 ppm among uremic patients on dialysis who died of a neurologic syndrome of unknown cause, among uremic patients on dialysis who died of other causes, and among control subjects, respectively. Mean brain concentrations of Al were significantly higher in both uraemic groups as compared to controls.	[82]

Abbreviations: ASD—Autism spectrum disorder; AUD—Alcohol use disorder; DE—Dialisys encephalopathy; MS—Multiple sclerosis.

## 3. Autism Spectrum Disorder

ASD is a neurodevelopmental disorder associated mainly with persistent deficits in social communication and repetitive, inflexible patterns of behavior. Depending on geographical region and diagnostic criteria, its prevalence in Asia, Europe, and the USA ranges from 0.2% to 2.5%. Worryingly, a significant increase in diagnosed ASD has been seen since the late 1990s [83], with a more than 20-fold increase between the 1970 and 2005 birth years in USA population [84]. Apart from a rise in global awareness of the subject of ASD and a more inclusive definition in the Diagnostic and Statistical Manual of Mental Disorders V (DSM V) [85], a real increase in ASD’s prevalence is being suggested. In 2014, Nevison [84] stated that in the United States since 1988, a real increase in ASD prevalence accounts for circa 75–80% of the tracked increase in its diagnosis. 

The pathogenesis of ASD is not fully elucidated [86]. Suspected risk factors of ASD are genetic factors, advanced parental age, prenatal infections, and exposure to toxic substances [83,85], among which some authors mention Al [87,88,89]. Noteworthy, over 1000 various substances were considered neurotoxic in laboratory studies, of which over 200 have been documented as neurotoxic in humans [90]. In USA population, exposure to some factors considered as risk factors of ASD represent constant (e.g., phthalates, atmospheric mercury (Hg)) or decreasing trends (e.g., lead (Pb), dioxins, vehicular emission), making them less likely to be involved in ASD prevalence in the USA [84]. 

To investigate the link between ASD and exposure to several metals, including Al, Sulaiman et al. [85] in 2020 published meta-analysis comprising case-control and cross-sectional studies. Apart from individual studies showing inconsistent results, the meta-analysis found that Al concentration in both hair and urine samples was positively correlated with ASD, while Al concentration in blood was negatively associated with ASD. Thus, a suggestion was made that ASD is presumably associated with impaired abilities of certain metal metabolism, detoxification, and excretion. Those authors supported the efforts to reduce lifespan exposure to neurotoxic metals, particularly in pregnant women and young children who are the most susceptible to their effects [85]. In 2022, Amadi et al. [86] published a meta-analysis of case-control studies on several toxic metal burdens in ASD patients. The results confirmed excessive toxic metal concentration in ASD patients [86]. The evidence of Al concentration in ASD sufferers’ brains is limited [86]. The first study conducted among 10 ASD donors’ brains reported elevated Al concentration with an intracellular and extracellular space (Table 4) [76]. However, McLachlan et al. [64] found no association was found between ASD and Al concentration in temporal gyri (Table 4). 

Among the sources of Al in infants, milk formulas, intravenous feeding solutions, and possibly Al-containing vaccine adjuvants are suggested [8]. However, the association between Al-containing adjuvants and ASD is highly controversial [91], as Al adjuvants are linked with minimal adverse effects [92,93]. In research by Mitkus et al. [94], Al diet and vaccine exposure during the first year of life did not exceed the minimal risk levels specified by ATSDR [95]. Additionally, no link between blood and hair Al and the history of immunization by Karwowski et al. [96] in a group of 85 healthy infants aged 9–13 months. However, authors of several studies emphasize the role of prospective epidemiological studies [91], with additional attention on exposure to metal [97]. 

## 4. Alcohol Use Disorder

Alcohol Use Disorder (AUD) is a chronic and progressive disease that affects users’ daily functioning. AUD is caused by the loss of control over the amount of alcohol consumed and the continuous need for alcohol consumption [98]. In AUD, Al accumulates in brain tissue, which can lead to dementia. This elevated Al accumulation can be caused by elevated permeability of the intestinal mucosa for Al, which is the result of excessive alcohol consumption [99]. Additionally, despite the fact that Al contained in beer should be removed by properly functioning kidneys, this applies only to moderate beer consumption, which does not occur in AUD [100]. 

Another beer ingredient, Si, inhibits the negative impact of beer on the human brain: Si significantly affects the bioavailability of Al and may reduce its neurotoxicity [101]. The hypothesis of the protective effect of Si was investigated in the research from 2004, which was carried out on mice. Mice were divided into three groups: the first group was given 2.5 mL commercial beer (5.5% volume) per week, the second group received 2.5 mL of silicic acid solution per week, and the third mice group received neither beer nor silicic acid. Next, after analysis, the levels of Al and Si in mice’s urine, feces, and brain were found, and it was proved that Si content in beer reduced Al uptake and its accumulation in the brain [102]. The other study also carried out on mice showed that Si contained in beer, the reason for the influence on Al toxicokinetics, can prevent inflammation and oxidative stress in the brain caused by Al [103].

Moreover, exposure to Al can be a risk factor for AD and dementia development, while Si contained in beer can protect against these diseases by decreasing of uptake of Al from the digestive tract and inhibiting its accumulation [104,105]. 

Beer not only affects Al bioavailability but also can reverse metal imbalance and pro-oxidative state that are caused by Al nitrate in the brain. In the study by González-Muñoz et al. [106], four groups of mice were studied: the first group was given deionized water, the second Al(NO_3_)_3_, the third Al(NO_3_)_3_ and silicic acid, and the fourth Al(NO_3_)_3_ and beer. As a result, in the third and the fourth group, a decrease in the concentrations of Al, Si, and thiobarbituric acid reactive substances (TBARS) and a decrease in the expression of TNFα, as well as an increase in the concentrations of copper (Cu), manganese (Mn), zinc (Zn), and antioxidants, compared to the second group. Those results suggested that Si reversed Al-induced influence to a significant extent [106]. Another study also confirms that beer reduces the oxidation processes in the brain, which are caused by the toxicity of Al. In this research, mice were divided into two groups: the first group was given Al(NO_3_)_3_ in drinking water, while the experimental mice were given Al(NO_3_)_3_ in combination with silicic acid or beer. It was observed that beer inhibits the decrease in the mRNA expression of endogenous antioxidant enzymes, prevents damage of lipids, and normalizes the expression of TNFα [107] (Table 5). 

These studies [102,103,104,105,106,107] refer to moderate alcohol consumption. In AUD, alcohol is consumed excessively. The concentration of Al and Si in the brain and liver of individuals with AUD was examined in a study from 2019. Brain and liver samples were collected in post-mortem examination from 31 patients with AUD and 32 patients without AUD as the control group. The study showed that AUD patients had elevated concentrations of Al in the brain (see Table 4). Moreover, the highest concentration of Al was detected in the frontal part of the thalamus, inferior longitudinal fasciculus, and frontal part of the insula. However, a higher concentration of Si was not observed in the brain of AUD patients, which suggests, that excessive consumption of alcohol results in significantly increased exposure to Al, but more studies are needed to investigate this in depth [77]. 

## 5. Multiple Sclerosis

MS is a chronic, autoimmunological disease causing demyelination of CNS. As its pathogenesis remains unclear, the search for its trigger factors remains a significant challenge in neurology [111]. Nowadays, exposure to Al is identified as a trigger factor. The study from 2018 carried out on brain tissue from 14 patients with MS revealed higher Al levels in both intracellular and extracellular locations [112]. In another study, to further show how high the concentration of Al is in the brain tissue of individuals with MS, Linhart et al. [113] compared the concentration of Al in the brains of MS patients with the control group of brains from non-MS patients. Although Al was detected in each donor, comparing Al concentrations proved that donors dying with a diagnosis of MS presented elevated Al concentrations [113]. Similar results were obtained by Exley et al. [78], whose study also suggests that the concentration of Al is significantly higher in the brain tissue compared to the control group of patients without several neurological diseases (AD, MS, and ASD—see Table 4) [78]. Another study pointed to the potential role of metabolic imbalance of Al in MS development: the concentration of Al in the scalp hair of patients with MS and healthy controls were examined. Results showed that the scalp hair Al concentration of MS patients was significantly lower, which can be caused by its accumulation in brain tissue and significant urinary excretion [79]. 

Patients with MS not only presented a high concentration of Al in brain tissue but also in the urine. Moreover, the concentration of Al excretion in individuals with MS was similar to those observed in patients undergoing metal chelation therapy [80]. Al excretion with urine seems of use in the non-invasive treatment of MS: therapy by Si-rich mineral water. In the study from 2017, carried out on a group of 15 patients with MS, the following regime was used: patients drank 1.5 L Si-rich mineral water daily per 12 weeks, which resulted in the increase of Al urinary excretion, which may consequently add to the reduction of its accumulation in the body, including the brain [114]. 

A link between exposure to Al and MS development gave rise to the inclusion of ethylenediaminetetraacetic acid (EDTA) chelation therapy in the treatment. The study from 2014 examined a group of patients with neurological disease; 85.6% (n = 101) of them had MS and a healthy control group. All patients were challenged with EDTA, and those who showed Al poisoning after this test were subjected to chelation therapy (EDTA iv once a week). The use of EDTA proved effective in removing excess Al. Additionally, shortening the duration of Al intoxication resulted in a significant improvement in the clinical condition of patients: there was a reduction in neurological disability and fatigue [115]. Fulgenzi et al. [116] described a case of a patient with MS treated by EDTA. After the challenge with EDTA, the level of Al in the patient’s urine was elevated. Next, the patient was treated by EDTA, and as a result, neurological condition improvement and remission of MS were all observed; additionally, the level of Al in the urine decreased to normal values [116].

## 6. Parkinson’s Disease

PD is a progressive neurodegenerative disorder manifested by motor symptoms such as slowness of movement, tremors, stiffness, postural instability, and non-motor symptoms: depression, anxiety, dementia, and autonomic dysfunction. The ultimate cause of PD is unknown, but studies point to risk factors such as age, family history, and pesticide exposure [117,118]. Additionally, long-term exposure to Al creates a risk factor for PD. Al accumulates in the substantia nigra and in Lewy bodies and disrupts the dopaminergic system by affecting the activity of the enzyme involved in the dopamine (DA) biosynthesis pathway [119,120]. Additionally, the research carried out on zebrafish showed that exposure to Al not only causes neurodegenerative processes but also influences the regulation of genes related to PD [2]. 

The main sources of Al are occupational exposure and environmental pollution. Occupational exposure to Al doubles the risk of PD [87]. Occupational exposure to Al as a risk factor for PD has been described in studies by Zeng et al. [121]. This retrospective study was conducted on a cohort of 37,000 male miners in Ontario and found that miners with respiratory exposure to Al had a 30% higher incidence of PD [121]. Moreover, the higher risk of PD seems to be correlated with the duration of exposure to Al. Additionally, the study by Martell et al. [122] showed that miners exposed to inhalation of finely ground Al dust had a significantly higher risk of developing PD compared to unexposed miners or the Ontario population [122]. It was ascertained that the combined exposure to Al and other metals increases their toxic effect on the –NS-Hg and has a synergistic effect with Al. The combination of Mn, Fe, and Al has been significantly associated with a higher risk of PD [123]. A similar relationship is exhibited between Al and pesticides: they accelerate the rate of formation of alpha-synuclein fibrils [124]. According to the study by Altschuler [125], another source of Al is constituted by Al-containing antacids which may be involved in PD development. Patients with peptic ulcer disease not only had an increased absorption of Al but also relied on Al-containing antacids [125]. 

The examination of the level of Al in the serum seems to be a valuable method in the diagnosis of early PD. An analysis of the level of Al in combination with disturbance in the elements’ homeostasis and inter-elements relationship by the neural-network algorithm can be a valuable method of early PD diagnosis: use of an artificial neuronal network (ANN) algorithm provides 95% accuracy [126]. Additionally, due to the role of Al in PD development, inhibition of its activity seems to be a therapeutic target. Al-induced neurodegeneration in PD can be restricted by the natural flavonoid—Quercetin. A study from 2016 conducted on rats showed that quercetin, used in the mechanism of reduction of Al-induced oxidative stress, largely inhibits neuronal apoptosis. For this reason, quercetin presents potential therapeutic value in slowing down the progression of PD [108] (Table 5). The other substance, curcumin, also seems to be useful in protecting the brain from Al-inducted neurotoxicity. The research carried out on rats showed that it inhibits disruption of the dopaminergic system in PD as a result of the normalization of activity of the tyrosine hydroxylase (TH), an enzyme involved in the dopamine biosynthesis pathway [109] (Table 5). The study from 2022 suggests that Centella Asiatica affects oxidative stress in the brain, inhibits neurodegeneration caused by Al, and presents potential therapeutic value in preventing Al-inducted PD [110] (Table 5).

## 7. Dialysis Encephalopathy

DE is a progressive, fatal disease linked to Al toxicity in CNS. Clinical manifestations of DE include dementia; language dysfunctions, such as slurred speech, stuttering, the problem with forming accurate phrases, permanent mutism, and aphasia; motor function damage, including myoclonus, epilepsy, tremor, flapping wing tremor, grimace, abnormal gait, athetosis, rigidity, and weakness; mental and behavioral disorders [127]. The main source of Al in DE is the water used to prepare dialysis fluid [128] and Al-containing phosphate binders, which were used for the prevention of hyperphosphataemia [129,130]. 

Al in DE accumulates in the brain, which can be observed in the postmortal examination. The study by McDermott et al. [81] of postmortal brain examination showed that the concentration of Al was elevated in some patients who deceased due to dialysis encephalopathy compared to the control group of dialysis and non-dialysis patients with uremia. Al accumulated mainly in the grey matter of patients’ brains. Moreover, the Al concentration in the patient’s brain remains elevated for up to four years after kidney transplantation [81]. Similar results were obtained by Alfrey et al. [82], who examined the concentration of Al in muscles, bones, and brains of uremic patients on dialysis. The study showed that the concentration of muscle and bone Al was higher as compared to the concentration in the control group. Additionally, the concentration of Al in the grey matter of the brain of patients with DE was higher compared to any of the control subjects or other uremic patients on dialysis [82]. Al accumulates in the neutrons and astrocytes of the cerebral cortex. The autopsy of three brains of patients who died of DE showed spongy lesions limited to the upper layers of the cerebral cortex, which contained vacuoles inside the neuropil and nerve cell bodies [131]. 

To reduce the risk of DE in patients with chronic kidney disease, reduction of exposure to Al is indispensable. Results of a study from 1980 showed that cleaning the dialysis fluid with a water softener, reverse osmosis, and deionizer can significantly reduce the development of DE incidence. Previously, seven patients had died of DE, and 16 of the 51 surviving patients had symptoms of DE. [132]. Additionally, some authors suggested that avoidance of Al-based phosphate binders and chelation agent treatments seem to be promising methods in the prevention of DE [133]. DFO is the chelation agent used in the Al intoxication of dialysis patients, which can also be used as a noninvasive method of identification of dialysis with Al overload. [134]. The combined use of DFO and hemodialysis was described as a good method of treatment for patients with severe Al encephalopathy. On the other hand, DFO cannot always be used in DE treatment: the high concentration of Al in serum after treatment with DFO induces the concentrations of toxic DFO-Al complexes and causes to worsen the condition of the patient. For this reason, at serum Al concentrations higher than 200 μg/L, DFO is not recommended for treatment [127,135].

## 8. Conclusions

The main source of exposure to Al is oral ingestion and inhalation, whereas the primary way of Al excretion is through urine. The literature clearly suggests that AD, AUD, MS, PD, and DE patients experience excessive accumulation of Al in the CNS. Epidemiological links between higher exposure to Al and their increased incidence have been observed in AD, PD, and DE. In AD and PD, the potential use of Al as a disease marker has been noted. Additionally, favorable results of the use of Al chelators were observed in AD, AUD, MS, and DE. Moreover, cleaning dialysis fluid from Al prevents the development of DE. The risk of Al toxicity via vaccination is minimal compared to the benefits presented by vaccinations alone, and data regarding skin Al absorption appear to be limited.

## Figures and Tables

**Table 1:** Examples of recommendations regarding aluminium exposure limits.

Source of Exposure to Al	Exposure to Al Limits	Comments	Organization, References
Occupational exposure limits	Al alkyls, NOS—PEL of 2 mg/m^3^; Al soluble salts—PEL of 2 mg/m^3^; Al metal and oxide (total dust)—PEL of 10 mg/m^3^; Al metal and oxide (respiratory fraction)—PEL of 5 mg/m^3^; Al pyro powders—PEL of 5 mg/m^3^; Al welding fumes—PEL of 5 mg/m^3^; Al stearate—PEL of 10 mg/m^3^; Al distearate—PEL of 10 mg/m^3^; Al tristearate—PEL of 10 mg/m^3^.	8-h TWA was used in this document.	Cal/OSHA [14,15]
	Al (total dust)—PEL of 15 mg/m^3^ Al (respirable fraction)—PEL of 5 mg/m^3^	-	OSHA [14]
	Al (total dust)—REL of 10 mg/m^3^; Al (respiratory fraction)—REL of 5 mg/m^3^;	Up-to-10-h TWA was used in this document.	NIOSH [14,16]
Oral exposure	TWI of 1 mg/kg bw/week		EFSA [17]
	NOAEL of 30 mg/kg bw/day; LOAEL of 50–75 mg/kg bw/day; PTWI of 2 mg/kg bw.	Major contributors to the total oral Al exposure were cereals and cereal-based products, accounting for 20–90% of total dietary Al exposure.	WHO [18]
Drinking water	For small water treatment facilities—0.2 mg/L For large water treatment facilities—0.1 mg/L	-	WHO [19]
	0.05 to 0.2 mg/L	Al level belongs to secondary standards, regarding substances that could cause “cosmetic effects (such as skin or tooth discoloration) or aesthetic effects (such as taste, odor, or color)”.	EPA [20]
Freshwater (regarding aquatic life)	CMC of 1–4800 μg/L CCC of 0.63–3200 μg/L	Wide range of Al CMC and CCC is caused by significant dependence of Al–bioavailability and certain factors (mostly important ones are total hardness, pH, and dissolved organic carbon).	EPA [21]
Toys	“Dry, brittle, powder-like or pliable toy material”—2250 mg/kg; “Liquid or sticky toy material”—560 mg/kg; “Scraped-off toy material”—28130 mg/kg.	SCHEER suggests that additional exposure from toys should be minimized due to high exposure to Al from other sources.	SCHEER [22]

Abbreviations: Al—aluminium; bw—body weight; Cal/OSHA—California’s Division of Occupational Safety and Health; CCC—criteria chronic concentration; CMC—criteria maximum concentration; EFSA—European Food Safety Authority; EPA—Environmental Protection Agency; LOAEL—lowest-observed-adverse-effect level; NIOSH—The National Institute for Occupational Safety and Health; NOAEL—no-observed-adverse-effect level; NOS—not otherwise specified; OSHA—Occupational Safety and Health Administration; PEL—permissible exposure limit; PTWI—provisional tolerable weekly intake; REL—recommended exposure limit; SCHEER—Scientific Committee on Health, Environmental and Emerging Risks; TWA—time-weighted average; TWI—tolerable weekly intake; WHO—World Health Organization.

**Table 2:** Examples of food and beverage content of aluminium.

Product	Mean Al Content	Reference
Cheddar cheese, sharp	3.9 ± 3.9 mg/kg	[23]
Beer ^a^	0.4–4.2 mg/L	[24]
Bread	1–14 mg/kg	[24]
Cocoa powder	80–312 mg/kg	[24]
Doughnut	9 ± 6 mg/kg	[23]
Flour	1–19 mg/kg	[24]
Fruit juice ^b^	0.4–47 mg/L	[24]
Herb-teas	14–67 mg/kg	[24]
Pancake mix	620 ± 460 mg/kg	[23]
Pasta	1–76 mg/kg	[24]
Wine ^c^	0.4–15 mg/L	[24]

Abbreviations: Al—aluminium; ^a^ and mixed drinks containing beer, draught beer; ^b^ and fruit juice drinks; ^c^ and fruit wine.

**Table 3:** Factors affecting gastrointestinal absorption of Al.

	Higher Absorption	Lower Absorption	Reference
pH	Acidic or alkaline	Neutral	[9]
Al compound	Al chloride, nitrate, citrate, lactate	Al hydroxide	[7]
Presence of other substances	Citrate, fluoride, maltol, lactate	Silicate, phosphate, polyphenol, sialic acid	[4]
Other factors	Larger amount of ingested Al	-	[12]
	iron deficiency in the diet		[7]

**Table 5:** Potentially neuroprotective agents for Al-induced diseases found in animal studies.

Disease	Animal Species	Neuro-Protective Agent	Neuroprotective Effect	Additional Information	Reference
AUD	mouse	Beer (Si)	Inhibition of Al-induced prooxidant and proinflammatory actions by decreasing TBARS levels and the expressions of GPx and TNFα and increasing the expressions of SOD (MnSOD and CuZnSOD) and CAT.	Other beer components possibly involved: alcohol, hop, polyphenols, and folic acid. Further studies considering the similar effects of non-alcoholic beer are needed. Harmful effects of alcohol consumption must be taken into account.	[107]
AD, PD	rat	Quercetin	Attenuation of neuronal death against Al-induced neurodegeneration by: - reduction of Al-induced oxidative stress, - prevention of Al-induced cyt c translocation, - up-regulation of Bcl-2, - down-regulation of Bax, p53, and caspase-3 activation, - reduction of DNA fragmentation, - attenuation of Al-induced mitochondrial swelling, loss of cristae, and chromatin condensation.	“Quercetin may be used as a prophylactic in order to slow down the progression of neurodegenerative diseases such as Alzheimer’s and Parkinson’s disease”.	[108]
PD	rat	Curcumin	Prevention of Al-induced DAergic neurotoxicity and related locomotor deficiencies (displayed by restored immunoreactivity of TH in SNc and VTA).	Curcumin could be considered as “a natural drug conferring the protection of the brain from heavy metals induced neurotoxicity”.	[109]
PD	rat	CAE	Alleviation of cognitive impairment, cellular damage, neurodegeneration, and cholinergic activity through attenuation of: - Al-induced oxidative stress (normalization of the MDA content and CAT and SOD activity in the cerebrum and cerebellum) - AChE activity in the cerebrum and cerebellum	CAE not only prevents but also reverses the aforementioned Al-induced negative effects. “CAE could be used as an antioxidant, anti-cholinesterase, memory enhancer, and neuroprotective agent”.	[110]

Abbreviations: AD—Alzheimer’s disease; Al—aluminium; AUD—Alcohol use disorder; Si—silicon; TBARS—thiobarbituric acid reactive substances; GPx—glutathione peroxidase; TNFα—tumor necrosis factor-alpha; SOD—superoxide dismutase; MnSOD—manganese SOD; CuZnSOD—copper-zinc SOD; CAT—catalase; Bcl-2—B-cell lymphoma 2; Bax—Bcl-2-associated X protein; PD—Parkinson’s disease; p53—tumor protein 53; cyt c—cytochrome c; DAergic—dopaminergic; TH—tyrosine hydroxylase; SNc—substantia nigra pars compact; VTA—ventral tegmental area; CAE—ethanolic extract of *Centella asiatica*; MDA—malondialdehyde; AChE—acetylcholinesterase.

## Data Availability

No new data were created or analyzed in this study. Data sharing is not applicable to this article.

## Abbreviations

AChE	Acetylcholinesterase
AD	Alzheimer’s disease
Al	Aluminium
ALS	Amyotrophic lateral sclerosis
ANN	Artificial neuronal network
ASD	Autism spectrum disorder
ATSDR	Agency for Toxic Substances and Disease Registry
AUD	Alcohol use disorder
Bax	Bcl-2-associated X protein
BBB	Blood-brain barrier
Bcl-2	B-cell lymphoma 2
BMP-2	Bone morphogenic protein 2
Bw	Body weight
CAE	Ethanolic extract of *Centella asiatica*
Cal/OSHA	California’s Division of Occupational Safety and Health
CAT	Catalase
CCC	Criteria chronic concentration;
CMC	Criteria maximum concentration
CSF	Cerebro-spinal fluid
CSN	Central nervous system
Cu	Copper
CuZnSOD	Copper-zinc SOD
cyt c	cytochrome c
DA	Dopamine
Daergic	dopaminergic
DE	Dialysis encephalopathy
DFO	Deferoxamine
EDTA	Ethylenediaminetetraacetic acid
EFSA	European Food Safety Authority
EPA	Environmental Protection Agency
Fe	Iron
GPx	glutathione peroxidase
Hg	Mercury
LOAEL	Lowest-observed-adverse-effect level
MDA	Malondialdehyde
Mn	Manganese
MnSOD	Manganese SOD
MS	Multiple sclerosis
NFTs	Neurofibrillary tangles
NIOSH	The National Institute for Occupational Safety and Health
NOAEL	No-observed-adverse-effect level
NOS	Not otherwise specified
OSHA	Occupational Safety and Health Administration
p53	Tumor protein 53
Pb	Lead
PD	Parkinson’s disease
PEL	Permissible exposure limit
PTWI	provisional tolerable weekly intake
REL	recommended exposure limit
SCCS	Scientific Committee on Consumer Safety
SCHEER	Scientific Committee on Health, Environmental and Emerging Risks
Si	Silicon
SNc	Substantia nigra pars compact
SOD	Superoxide dismutase
TBARS	Thiobarbituric acid reactive substances
Tf	Transferrin
TfR	Transferrin receptor
TGF-ꞵ1	Transforming grow factor beta 1
TH	Tyrosine hydroxylase
TNFα	Tumor necrosis factor alpha
TWA	Time weighted average
TWI	Tolerable weekly intake
VTA	Ventral tegmental area
WHO	World Health Organization
Zn	Zinc
